# Thermal Properties and Drying Shrinkage Performance of Palm Kernel Shell Ash and Rice Husk Ash-Based Geopolymer Concrete

**DOI:** 10.3390/ma17061298

**Published:** 2024-03-11

**Authors:** Mohd Na’im Abdullah, Faizal Mustapha, Nurul ‘Izzati Yusof, Tabrej Khan, Tamer A. Sebaey

**Affiliations:** 1 Department of Aerospace Engineering, Faculty of Engineering, Universiti Putra Malaysia, Serdang 43400, Malaysia; faizalms@upm.edu.my (F.M.);; 2 Engineering Management Department, College of Engineering, Prince Sultan University, P.O. Box 66833, Riyadh 11586, Saudi Arabia; tsebaey@psu.edu.sa; 3 Mechanical Design and Production Department, Faculty of Engineering, Zagazig University, Zagazig 44519, Egypt

**Keywords:** geopolymer, thermal, drying shrinkage, palm kernel shell, rice husk ash, concrete

## Abstract

This study aims to develop suitable formulations of geopolymer concrete (GPC) by varying the percentages of the geopolymer with aggregates and evaluating the performances in thermal and mechanical properties of palm kernel shell ash (PKSA)-GPC compared to rice husk ash (RHA)-GPC and ordinary Portland cement concrete (OPCC). Preliminary tests were conducted to select the best mix design ratios before casting the specimens. Then, the performance of the PKSA-GPC, RHA-GPC and OPCC specimens was evaluated based on their thermal performance and drying shrinkage. The mix designs of PKSA-GPC 70:30, PKSA-GPC 60:40, PKSA-GPC 50:50 and PKSA-GPC 66.6:33.3 were found to produce an acceptable consistency, rheological and thixotropic behaviour for the development of the GPC. PKSA-GPC showed a better thermal performance than the RHA-GPC and OPCC due to their strong and dense intumescent layers and slow temperature increment upon exposure to a high flame temperature from ambient temperature to 169 °C. The low molar ratio of the Si/Al present in the PKSA-GPC created a thermally stable intumescent layer. In the drying shrinkage test, PKSA-GPC 60:40 and RHA-GPC 60:40 shared an equal drying shrinkage performance (5.040%) compared to the OPCC (8.996%). It was observed that microcrack formation could significantly contribute to the high shrinkage in the PKSA-GPC 50:50 and RHA-GPC 70:30 specimens. The findings of this study show that PKSA could be incorporated into GPC as a fire-retardant material due to its capability of prolonging the spread of fire upon ignition and acting as an alternative to the conventional OPCC.

## 1. Introduction

Concrete is one of the most essential construction materials that is used for building applications. It comprises cement, aggregates and water. It is reported that the consumption of concrete is the second highest on the earth, immediately after water [1]. This is due to the high amount of concrete produced every year. The production of concrete causes a detrimental impact on the environment [2]. For instance, acidification and eutrophication are several environmental consequences of concrete production, apart from global warming [3]. This is because of the cement used in the production of concrete. It is reported that cement production contributes to 5–8% of global carbon dioxide emissions [4]. Thus, there is an urgent need to replace ordinary Portland cement (OPC) concrete with cementless concrete. Many studies have discovered that the replacement of OPC concrete with geopolymer concrete (GPC) could reduce the carbon dioxide emissions caused by OPC concrete production [5,6]. Moreover, the usage of geopolymer concrete as thermal insulation can also reduce the high energy consumption of buildings and in construction, as compared to OPC concrete [7]. This is because of the high water retention and low thermal conductivity of GPC, caused by its porous nature. Furthermore, there has been a significant increase in the number of high-rise buildings in Malaysia where concrete is mainly used in their foundations. This is because concrete is generally classified as a non-flammable material. However, fires could significantly impact the structure of this concrete as fires have a high tendency to cause explosive spalling [8]. Explosive spalling happens when concrete is subjected to high temperatures in which the water vaporises more rapidly than it can escape. This phenomenon leads to an increase in the vapour pressure inside the concrete. In the worst-case scenario, the build-up of the vapour pressure might cause the concrete to explode.

GPC is a type of concrete formed from the reaction of aluminium and silicate-containing compounds with the presence of an alkaline activator. Many studies have been conducted to compare the performance of GPC with OPC concrete [9,10,11]. These studies have found that GPC has an outstanding performance in compressive strength, flexural strength, tensile strength and modulus of elasticity compared to OPC concrete. Nikoloutsopoulos et al. (2021) conducted a study to compare the physical and mechanical properties of fly-ash-based GPC and OPC concrete. The researchers found out that compared to OPC concrete, the GPC exhibited competitive compressive strength after achieving its maximum strength after three days, which remained constant even after two years. In addition, the compressive strength of the GPC fulfilled the EN 206-1 standard [12] with values of 33.1, 45.3 and 43.8 MPa for a fly ash content of 375, 563 and 750 kg/m^3^, respectively. In addition, the tensile strength of the geopolymer concrete was within the range specified by Eurocode 2 and obtained about 50% less tensile strength than the OPC concrete. This study also reported that the ratio of the binder (fly ash) to aggregates contributes a significant effect to the mechanical properties of the GPC [13]. Yang et al. (2023) claimed that beams constructed from the combination of a geopolymer sea-sand concrete (GSSC) and basalt fibre-reinforced polymer (BFRP) exhibited a greater ultimate load capacity and smaller ultimate deflection due to the use of MgO as an expansion agent which reduced the concrete shrinkage [14]. Neupane (2018) also claimed that the high durability possessed by GPC was one of its most outstanding advantages compared to conventional OPC concrete. This is due to the difference in the binding system, where it does not rely on calcium compounds for the formation of the matrix [15]. In addition, the calcium oxide present in the OPC is very susceptible in an acidic environment and prone to sulphatic attack [16]. Many experimental results on the durability of GPC that other researchers had conducted previously proved that GPC has a high resistance against aggressive environments. For example, Azarsa and Gupta (2020) conducted a study to assess the chemical leachability and durability of metals from GPC paver blocks and OPC paver blocks over 150 and 240 days of exposure. The results revealed that the pH of GPC paver blocks is 13.6% more alkaline than the OPC paver blocks. This is because of the presence of potassium hydroxide in the geopolymer concrete [17]. In a study by Yang et al. (2023), GPC demonstrated excellent resistance to seawater corrosion. The results showed that the strength corrosion resistance coefficients of GPC after 360 days of immersion in water and seawater were 0.06 and 0.085, respectively [18].

The green materials present in GPC are mainly waste materials from fly ash, ground granulated blast slag (GGBS) and rice husk ash. Moreover, palm oil biomass can also be utilised as a green material for geopolymer concretes due to the rich compositions of silica and aluminium present in the biomass. The utilisation of these green materials in concretes can eventually lead to a cleaner environment, as the production of these concretes produces 54% less carbon dioxide emissions and six times lower energy consumption than conventional OPC concrete [19]. Malaysia is among the world’s most prominent and biggest palm oil exporters. Almost 90% of the fresh fruit bunches in a palm oil mill are converted into agricultural by-products during the pruning, replanting and milling processes of oil palm. The palm oil industry currently generates an alarming amount of waste, with approximately 90% of the by-products from palm oil processing being classified as waste and referred to as palm oil biomass [20]. Among the wastes obtained from palm oil production are palm kernel shell (PKS), empty fruit bunch (EFB), oil palm trunk (OPT), oil palm shell (OPS), palm oil fuel ash (POFA) and oil palm frond (OPF). The accumulation of these wastes creates an environmental concern as the waste is discarded through incineration in the mills or dumped haphazardly, leading to air and land pollution [21]. This suggests that a significant portion of the resources involved in this industry are not fully utilised and remain unused. Hence, numerous ways have been newly developed to incorporate palm oil biomass into the existing materials to upgrade the values and properties of the materials. Recently, researchers have been trying to incorporate simple additive compounds originating from palm oil biomass in building materials owing to their low cost and sustainability to enhance the properties of the building materials. Adnan et al. (2019) conducted a study to replace cement with POFA in concrete brick since brick is one of the fire-retardant materials available in the industry. This replacement was proposed due to the pozzolanic properties possessed by POFA. The authors believed that the study could reduce carbon dioxide emitted by the cement in the concrete brick. In addition, the incorporation of POFA can influence the thermal and mechanical strength of the brick. The findings of their study revealed that the compressive strength decreased with the increase in POFA incorporated in the brick [22]. These results demonstrated that the incorporation of POFA in a brick did not improve the mechanical properties of the fire-retardant material. However, the thermal properties of the concrete brick had significantly improved with the presence of POFA incorporated in the material.

Moreover, Abdullah and Hussin (2010) conducted similar research to incorporate POFA into cement-based aerated concrete. Similar results were obtained where improved thermal properties of the concrete were achieved by incorporating the POFA into the materials. These studies have shown some similarities in that the addition of palm oil biomass into the fire-retardant material managed to influence the thermal and mechanical strength of the material [23]. In addition, Jong and Teo researched concrete containing POFA and PKS to investigate the effect of the elevated temperature on the concrete that contains POFA and PKS. The findings of the study showed that the concrete containing POFA and PKS could withstand an elevated temperature. In other words, the thermal properties of the concrete containing POFA and PKS were improved. Nonetheless, the compressive strength of these concretes decreased when subjected to high temperatures [24]. On the other hand, research carried out by Sulaiman et al. showed quite different results on the mechanical properties of the concrete incorporated with oil palm ash, which showcased better compressive strength than conventional concrete. The study discovered that 10% sand substitution with ash yielded a 16% increment in compressive strength compared to the control concrete, with a value of 39.45 MPa. Moreover, the study showed that a high percentage of oil palm ash mixed with the concrete gave better performance on its compressive strength [25]. Numerous research studies have been completed on the utilisation of palm oil biomass waste, especially to incorporate various types of geopolymer into the composites of industrial building materials, including concrete and bricks. For example, Tam et al. conducted a study incorporating fly ash and slag as a cement partial replacement in GPC [26]. However, most of the studies conducted have focused on the mechanical properties of the materials, such as compressive strength and tensile strength, while limited information and studies were conducted on the fire and the physical properties of the materials, such as water absorption and drying shrinkage. 

This research aims to develop novel and optimum formulations of GPC for fire resistance by varying the percentages of the geopolymer loads with the aggregates. GPC is developed by incorporating palm oil biomass, specifically PKS, which comprises a high amount of silica and alumina as the aluminosilicate binder to bind the aggregate materials together and form a solid matrix. The stable oxides present in PKSA offer excellent fire-retardant properties when incorporated into GPC. In this study, the performance of the fire-resistant GPC material was evaluated through physical and microstructural analysis. Then, its performance was compared with rice husk ash (RHA) geopolymer concrete and conventional OPC concrete. The development of geopolymer concrete from the utilisation of palm oil biomass can help mitigate the risk of pollution caused by the accumulation of these wastes.

## 2. Materials and Methods

### 2.1. Raw Materials

The PKSA from the palm oil biomass used in this study was provided from BAC Biomass, Kampung Gajah, Perak, Malaysia. The ash obtained was the by-product waste from the solid fuel combustion, which underwent combustion at 600 °C from a boiler. At the same time, the RHA in this study was obtained from Maerotech Solution Sdn Bhd, Nilai 3, Negeri Sembilan, in the form of refined ash. The chemical composition of the PKSA and RHA from X-ray fluorescence (XRF) analysis is shown in Table 1.

Furthermore, the OPC in this study was obtained from YTL Cement to be used as the raw material for the control concrete. The OPC is a standard binder used in concrete production. Moreover, the sodium hydroxide utilised in this study was supplied by R&M Chemicals (Petaling Jaya, Selangor, Malaysia) in white pellet form with a molecular weight of 40.00 g/mol. This study employed 14 M of sodium hydroxide solution as a higher concentration of sodium hydroxide possessed higher mechanical and thermal properties compared to 6–12 M [27].

The sodium silicate was supplied by R&M Chemicals (Petaling Jaya, Selangor, Malaysia) in the form of a very viscous liquid. The mixture of sodium hydroxide and sodium silicate produced an alkaline-activated solution used to dissolve the silica and alumina compound present in the PKSA and RHA. Lastly, the sand acquired from a local source was used as the fine aggregate in this study at approximately 0.10–0.25 mm.

### 2.2. Ash Preparation

**Figure 1:**
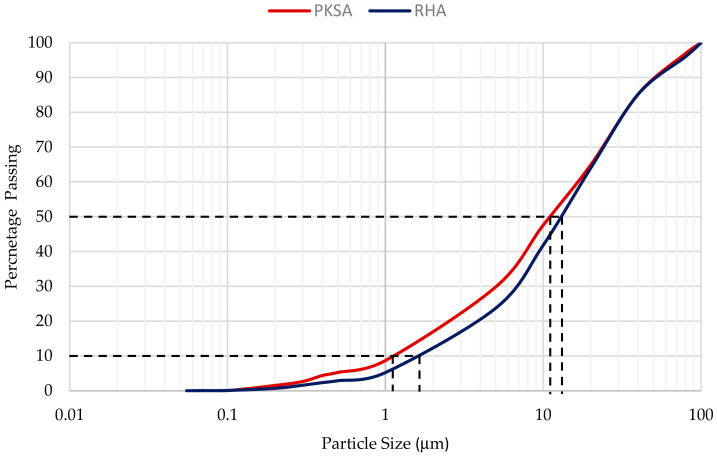
Particle size distribution for PKSA and RHA.

After obtaining the raw PKS Ash from BAC Biomass, 2.5 kg of raw PKS Ash was ground in a Pulverizing Machine RT-02A (Mill Powder Tech, Tainan, Taiwan) grinder. After the grinding process, the ground PKS ash was sieved with Endecotts Laboratory test sieve (Endecotts, London, UK) according to ASTM E11 standards [28] to remove unwanted impurities. In addition, this process was conducted to ensure uniform particle size distribution of the PKS ash before proceeding with the casting of the geopolymer concrete specimens. PSD for PKSA ranged from 0.067 μm to 56.63 μm, with 50% of particles finer than 10.75 μm and 10% of particles finer than 1.24 μm, as shown in Figure 1. The specific surface area was 2.4164 m^2^/g. A similar procedure was performed for the raw RHA. PSD for RHA ranged from 0.061 μm to 84.5 μm, with 50% of particles finer than 14.76 μm and 10% of particles finer than 1.67 μm, as displayed in Figure 1. The specific surface area was 1.5562 m^2^/g. The ash material is said to disperse more easily when it has a higher specific surface area, which in this case is the PKSA.

### 2.3. Geopolymer Concrete Preparation

**Figure 2:**
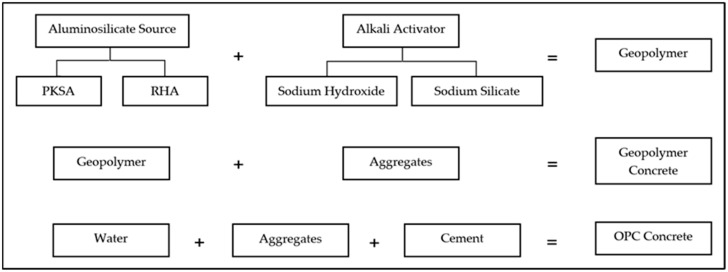
General formulation for the formation of GP, GPC and OPCC.

OPC concrete is a structural material consisting of OPC bonded with water and aggregates. The standard aggregates used for producing concrete are sand and gravel. The general formulation for the formation of the geopolymer, geopolymer concrete and the formation of OPC concrete is presented in Figure 2. 

With reference to the study conducted by previous researchers [29,30], the ratio used for the aluminosilicate source to alkaline activator (AA) solution was 2.5, while the ratio used for the sodium hydroxide solution and sodium silicate solution for the AA was 5.5. The chemical compositions of the mixture for these specimens are shown in Table 1. A total of 48.36 g of sodium silicate solution was mixed with 8.78 g of sodium hydroxide solution in an IKA RW 20 digital mechanical stirrer under constant stirring for 5 min. Next, 10.4 g of PKSA was gradually added to the mixture under constant stirring. Then, 60 g of sand was added gradually to the mixture under constant stirring. The rpm of the digital stirrer was measured when the mixture obtained a good consistency. The mix ratios designed for the PKSA-GPC and RHA-GPC are shown in Table 2.

**Figure 3:**
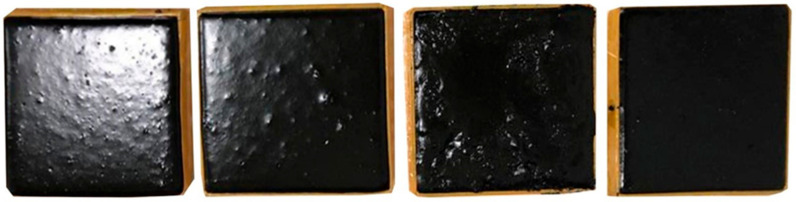
GPCC specimens.

The basic materials for the development of geopolymer concrete were aluminosilicate source, fine aggregates, coarse aggregates and AA. The concrete control specimens were prepared by mixing the cement, sand and gravel. The standard ratio for the concrete preparation was cement/fine aggregate/coarse aggregate with a ratio of 1:1:2 on a weight basis. Several studies conducted by previous researchers have found that aggregates only influence the strength of the concrete and not its other properties [31,32]. Therefore, 1:1 was used as the cement to sand ratio on a weight basis. The gravel was opt-out for the small specimens as the analyses focused on other performances of the concrete and did not emphasise the strength of the concrete. Next, the ratio of water to cement in this study was 0.45. The specimens were prepared by mixing 300 g of sand with 300 g of OPC in a cement bucket. Then, 135 g of water was added gradually to the mixture of sand and OPC. The mixture was then mixed until a well-mixed mixture was obtained. The mixture was transferred into aluminium moulds with the size of 100 mm × 100 mm × 15 mm in accordance with ASTM E119-19 Standard Test Methods for Fire Tests of Building Construction and Materials [33]. Prior to the experiment, the aluminium moulds were coated with two layers of paint to protect them against corrosion. Figure 3 illustrates the GPCC specimens after drying for 48 h at room temperature.

### 2.4. Design of Experiments

In this study, the ratio of geopolymer to aggregates was manipulated (90:10, 80:20, 70:30, 66.6:33.3, 60:40, 50:50 and 40:60) while the other variables remained constant. These variables were the ash-to-alkali ratio, curing temperature, sodium hydroxide-to-sodium silicate solution weight ratio and the molarity of the sodium hydroxide (14 M). The design of experiments for the PKSA-GPC and RHA-GPC specimens is shown in Table 3.

### 2.5. Direct Flame Test

The performance of the specimens when subjected to a high flame temperature was studied according to the ASTM E119-19 Standard Test Methods for Fire Tests of Building Construction and Materials [33]. A propane air burner with a maximum temperature of 1100 °C and a maximum heat flux of 106 kW/m^2^ was used for the direct flame test.

**Figure 4:**
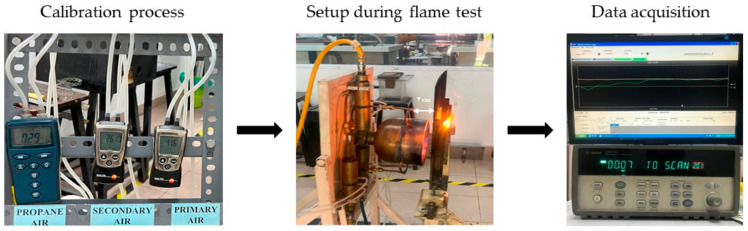
Direct flame test process.

Prior to the experiment, the propane air burner was calibrated by setting up the airflow for the primary and secondary air. The propane gas was maintained at 290 Pa, while the pressure for the primary and secondary airflow was maintained at 4160 Pa and 2610 Pa, respectively. The temperature was measured using a Type-K thermocouple connected to a DAQ sensor with an average temperature of 1100 °C. The nozzle of the propane air burner was placed 7 cm from the surface of the specimen. A Type-K thermocouple was attached to the non-exposed side of the specimen to measure the temperature at that point. To ensure a uniform temperature during the experiment, a Type-K thermocouple was also placed on the front side of the sample. The temperature was recorded at every 5 min interval, starting from room temperature up to 1100 °C. In addition, a time–temperature graph was plotted, while the thickness of the intumescent layer was observed and measured. The direct flame test process is shown in Figure 4.

### 2.6. Thermogravimetric Analysis (TGA)

Thermogravimetric analysis (TGA) is a method that determines the changes in the weight of the materials based on the temperature change. The TGA functions by heating the mixture at a high temperature until it decomposes into gas. The results of the TGA can determine the thermal stability of a specimen. The TGA was performed with the TGA-DSC HT 3, model Mettler Toledo (Columbus, OH, USA) using 20 mg of specimen mass. The analysis was carried out in an inert atmosphere and synthetic air at a temperature of 25–900 °C with a heating rate of 20 °C/min.

### 2.7. Shrinkage Test 

The drying shrinkage test was carried out to determine the moisture loss in the geopolymer concrete, which was performed according to the ASTM C426-16 Standard Test Method for Linear Drying Shrinkage of Concrete Masonry Units [34]. The lengths of the OPC concrete and the geopolymer concrete were measured in an interval of 1 day for 14 days. The graph of the percentage of shrinkage against days was plotted. The drying shrinkage was calculated using Equation (1):

(1) $$S_{L}\%=\frac{L_{0}-L}{L_{0}}\times 100$$

where

$S_{L}=$ shrinkage length;
$L_{0\;}=$ initial length;
L = length at the given time.

## 3. Results and Discussions

### 3.1. Geopolymer Concrete Preliminary Design Characteristic

Several ratios of geopolymer (GP) and fine aggregates (sand) were tested, while the performance of each ratio was evaluated. Table 4 summarises the observations made during the best mix design ratio selection.

The mixture became warmer during the mixing of these ashes with the AA, indicating the heat released by the mixture to the surroundings. It shows that this process possesses an exothermic reaction. In addition, this condition showed that dissolution occurred in the mixture during the geopolymerization process. Xu and Van Deventer (2001), who studied the geopolymerization of aluminosilicate minerals (containing source materials such as building residues, fly ash, furnace slag and pozzolan), stated that the process of geopolymerization starts with the Si-O bonds broken by the reaction with OH⁻. When the Si-O bonds are broken, silicate and aluminosilicate species are released into solution as oligomers, allowing for mobility and hydrated reaction products with NaOH and hence forming the [M_x_(AlO_2_)_y_(SiO_2_)_z_·*n*MOH·*m*H_2_O] gel [35]. For the thermodynamic model, Provis (2006) proposed the (MAlO_2_)(SiO_2_)_x_ model, where M is an alkali metal cation. The energetic basis of chemical ordering in geopolymers is due primarily to the exothermicity of [≡Si-O-Si≡] + [≡Al-O-Al≡] ⇔ 2 [≡Si-O-Al≡]. The energetics of this reaction depend on several factors, including the cations present and the positions of the centres within the network structure [36]. However, Bosenick et al. (2000) mentioned that bond ordering energies in minerals (Si and Al) are insensitive to composition provided that the variation is only in the ratio of the ordering cations, and so any possible dependence of cations present and the positions will not be considered [37]. Previous studies conducted by Ling et al. and Mohamed et al. also proved that the dissolution reaction was exothermic [38,39]. Ling et al. (2019) reported that two major exothermic peaks were observed in a calorimetric curve using a semi-isothermal calorimetry device. The first peak indicated the dissolution process, while the second peak indicated the polymerisation peak, respectively [38]. Moreover, Mohamed et al. (2019) reported a high amount of heat released during the dissolution process with a value of −43.5067 J/g by using a class C fly ash to AA ratio of 2.5, whereby the ratio used was similar to the ratio used in this study [39].

Once the GP mixture was mixed with the sand, the mixture became cooler. Dissolution of reactive components in the aggregates into the AA solution can be an endothermic process as it involves the absorption of heat. Aggregates in concrete typically contain reactive minerals like calcium silicates or calcium aluminate. When these aggregates come into contact with the alkaline activator solution present in the geopolymer mixture, some of their components dissolve into the AA solution [40]. This scenario might happen as the gelation, polymerisation and hardening of the GP matrix began to occur. Furthermore, water evaporation from the aggregate surfaces during curing can also contribute to an endothermic effect by absorbing heat from the surroundings, resulting in the evaporation of free water. The water molecules in the mixture absorbed energy from the surroundings to recover the energy lost during evaporation. Thus, the absorption of heat from the surroundings caused the mixture to cool down. Moreover, the addition of silica from the sand promoted the geopolymerization rate [41], and hence increased the evaporation rate of the free water present in the GP mixtures. 

During the mixing process, it was observed that all the mix ratio mixtures had a good consistency except for the GP to the sand ratio of 40:60 as the mixture turned into a clump. This condition was due to the high volume of sand present in the mixture. The behaviour of the mixtures during the specimen casting can be observed in Table 4. The mixtures with the ratios of 90:10 and 80:20 were too diluted and watery due to the high volume of GP compared to the sand present in the mixture. On the other hand, the mixture with the ratio of 40:60 behaved like a solid as it clumped, as discussed previously. Moreover, all the mixtures with the ratios of 70:30, 60:40, 50:50 and 66.6:33.3 showed rheological properties, portraying thixotropic behaviour. This behaviour could be seen when the mixtures solidified when undisturbed but turned into a thick fluid when stirred or agitated before proceeding with the curing process. Additionally, the flow of the fluid mixtures did not flow smoothly when poured into the moulds for specimen casting due to their thixotropic behaviour. Thus, the mix ratios of 70:30, 66.6:33.3, 60:40 and 50:50 can be used as the finalised mix design for GPC in this study owing to their consistency, rheological and thixotropic behaviour.

### 3.2. Thermal Performance of Geopolymer Concrete

The thermal performance of GPC was analysed from the physical changes in the sample due to elevated temperature, fire resistance performance, thermal expansion, thermal stability and microstructural properties.

#### 3.2.1. Physical Changes Due to Elevated Temperature

**Figure 5:**
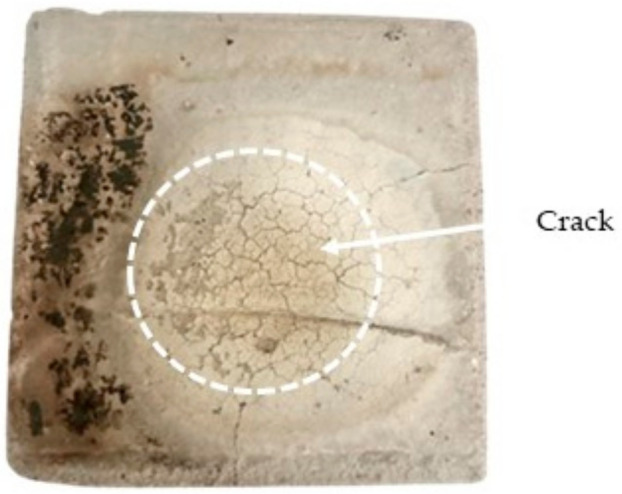
OPCC after exposure to high temperature.

**Figure 6:**
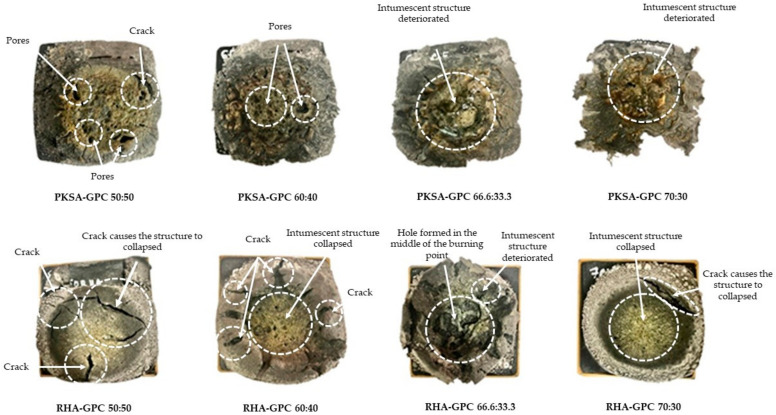
PKSA and RHA-GPC after exposure to high temperature.

A direct flame test was conducted to determine the performance of the OPC concrete (OPCC), PKSA-GPC and RHA-GPC upon direct exposure to a high flame temperature. Figure 5 displays the physical changes in the OPCC, while Figure 6 depicts the physical changes in the PKSA-GPC and RHA-GPC after being subjected to high temperatures for 60 min. Based on Figure 5 and Figure 6, numerous cracks were apparent on the surface of the OPCC and the GPC. Moreover, all the GPC mix ratios experienced expansion.

The cracks formed on the OPCC were due to the sudden increase in temperature. Upon exposure to the high flame temperature, the aggregates and the OPC paste started to expand. These expansions are known as thermal expansion, contributing to macrocracking in the concrete [42]. During the third minute of the fire testing, during which the temperature was around 150 °C, drips of water drops were noted along the surface of the concrete structure where the surface temperature was the temperature taken at the surface of the GPC when the GPC was subjected to a high flame temperature. On the other hand, the non-exposed temperature was the temperature taken at the other side (backside) of the GPC, where the surface was not subjected to any high flame temperature. The occurrence of the water dripping showed that the free water present in the structure started to evaporate and caused the cement paste to shrink. In addition, this process occurred rapidly due to the water expansion caused by the increase in temperature [43]. This process resulted in weak cohesive forces between the water molecules. Thus, the dripping of water occurred due to the massive escape of water vapour from the OPCC.

As the temperature increased, the aggregates present in the concrete kept on expanding while the OPC paste continued to shrink. As these processes opposed each other, microcracks started to occur. The cracks started to be larger and more obvious during the ninth to the twentieth minute of the testing with the surface temperature between 500 and 800 °C, indicating that the hydration components, such as calcium hydroxide and calcium silicate hydrate present in the OPC paste, began to decompose. Hence, large cracks were observed, as presented in Figure 5. Moreover, a significant reduction in the mechanical strength of the concrete will occur from the decomposition and the dehydration of these components [44,45]. Hence, the concrete samples should be removed and replaced as they will influence the strength and safety of a structure.

Meanwhile, all the GPC underwent expansion upon exposure to high flame temperature. The surface of PKSA-GPC 50:50 demonstrated numerous cracks and pores, and the intumescent layer of the sample collapsed due to the cracks on the sample surface during the flame test. In the meantime, the intumescent layer for samples PKSA-GPC 66.6:33.3 and PKSA-GPC 70:30 started to deteriorate after 37 min during the flame test. The deterioration causes a rapid increase in non-exposed temperature. In contrast, the intumescent layer for PKSA-GPC 60:40 was fully intact, and no deterioration occurred during the flame test with minimal pores on the surface of the sample. 

**Figure 7:**
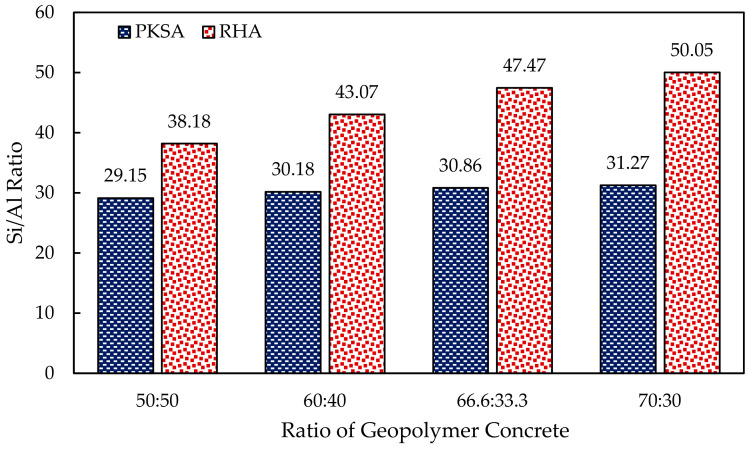
Molar ratio of Si/Al in the GPC.

Unlike the PKSA-GPC, all the RHA-GPC formed holes and cracks on the surface during the fire resistance test. RHA-GPC 66.6:33.3 started to fail after 20 min of the fire resistance test. The holes and deteriorated intumescent structure failed to give any protection for the non-exposed part of the GPC. Further, large cracks and holes on 45 min of the fire resistance test caused the intumescent structure to collapse for RHA-GPC 70:30. The formations of these holes were due to the failure during the intumescent process caused by the high-water content present in the GPC from the incomplete geopolymerization process. When subjected to high temperatures, the surface of the GP began to expand and formed a porous structure known as the intumescent layer. Since the RHA-GPC was rich in alumina and silica, as observed in Figure 7, the reactions in the GP matrix occurred at a fast rate. Moreover, the high molar ratio of Si/Al in the RHA-GPC also contributed to the increased reaction rate in the RHA-GPC matrix.

The water vapour present in the GPC rapidly evaporated and acted as the blowing agent, creating a foam layer that absorbed heat to protect the substrate. Moreover, the water vapour travelled from the hotter surface to the cooler inner part of the structure due to the temperature gradient. As the temperature at the surface of the GPC increased, the water molecules gained more energy due to the collision of the molecules, creating a fast evaporation rate and an increase in the matrix pressure. The cracks observed on the surface of the GPC were due to the high matrix pressure. The fast evaporation rate led to a fast expansion of the pores of the GPC. This scenario caused the GP matrix to become weaker, allowing the water vapour to escape from the GPC [46]. Since the intumescent process was unable to occur completely and crystallised in time, heat and fire were able to pass through the GP matrix and form a hole.

Based on Figure 6, highly dense and compact intumescent layers were observed on the surface of the PKSA-GPC, which might be due to the low molar ratio of the Si/Al present in the PKSA-GPC, as shown in Figure 7. This was because the low molar ratio of the Si/Al produced a very rigid 3-dimensional polymer network [47]. Hence, the intumescent layers could develop effectively and be able to undergo proper crystallisation, which in turn created a thermally stable intumescent layer. In conclusion, PKSA-GPCs had better fire performance than the OPCC and RHA-GPC, as the RHA-GPC underwent an incomplete intumescent and crystallisation process. In addition, the cracks on the surface of the OPCC demonstrated that the structural strength of the OPCC had deteriorated completely at 500–800 °C as discussed previously, while the compact intumescent layers of PKSA-GPC were thermally stable, which could delay the spread of fire longer than both OPCC and RHA-GPC. 

#### 3.2.2. Fire Resistance Performance of Geopolymer Concrete 

**Figure 8:**
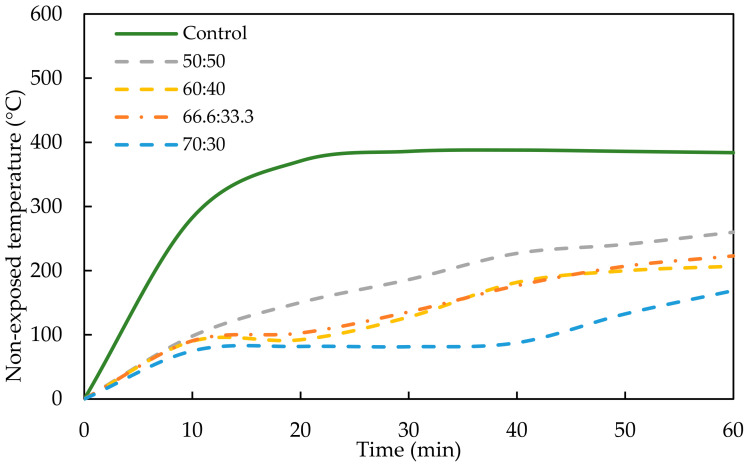
Thermal performance of PKSA-GPC.

**Figure 9:**
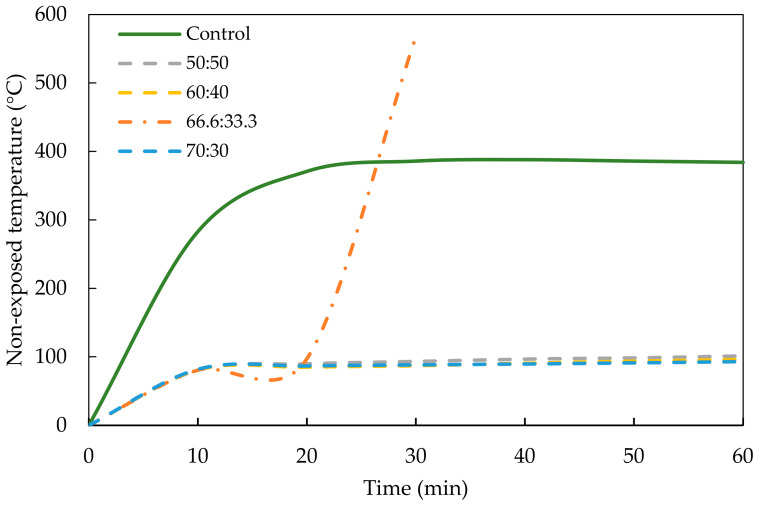
Thermal performance of RHA-GPC.

The thermal development of the OPCC and the GPC mix ratios could be determined by measuring non-exposed temperature from the direct flame test experiment. Figure 8 and Figure 9 show the thermal development of the OPCC, PKSA-GPC and RHA-GPC with different mix ratios of GP to sand. 

Based on both figures, the OPCC acted as the control concrete that experienced a high increment of non-exposed temperature compared to the PKSA-GPC and RHA-GPC. Figure 8 and Figure 9 show a sharp increase in non-exposed temperature for the OPCC, PKSA-GPC and RHA-GPC in the first 10 min upon being subjected to a high flame temperature. Both figures reveal that the non-exposed temperature for OPCC increased significantly from ambient temperature to 283 °C. On the other hand, Figure 8 displays that the temperature of the PKSA-GPC with a mix ratio of 70:30 had the lowest increment in temperature from ambient temperature to 75.1 °C. In contrast, the PKSA-GPC with a mix ratio of 50:50 had the highest increment in non-exposed temperature, which rose to 98.1 °C from the ambient temperature. Next, Figure 9 depicts no distinct trend for the increment of the non-exposed temperature of the RHA-GPC in which the temperatures increased to approximately 81.2 ± 0.6 °C. Based on these observations, the OPCC had the highest temperature increment, followed by PKSA-GPC 70:30, RHA-GPC and PKSA-GPC 50:50, consecutively. 

A rapid increase in the non-exposed temperature of the OPCC in the first 10 min was due to the chemical reaction in the OPCC upon exposure to a high flame temperature. In this period, ettringite formation occurred, resulting in the debonding of the cement paste from the aggregates, swelling of the hardened concrete, an increase in the capillary porosity and lessening the cohesiveness of the cement paste [48]. Unlike OPCC, the rapid increment in the non-exposed temperature for the PKSA-GPC and RHA-GPC was caused by the formation of an intumescent layer where expansion began to occur due to the reaction of the aluminosilicate source and AA present in the GPC. Next, the non-exposed temperature of OPCC experienced a constant temperature after the twentieth minute at the non-exposed temperature of approximately 350 °C, as shown in Figure 8. This event occurred as the dehydration and the decomposition of the hydration components took place, as previously discussed in Section 3.2.1. 

In addition, Figure 9 displays that the non-exposed temperature of all mix ratios of RHA-GPC remained constant after the 10th minute except for the RHA-GPC with a mix ratio of 66.6:33.3. The intumescent layer for RHA-GPC 66.6:33.3 started to expand rapidly after the 10th minute from 1.4 cm to 2.2 cm and helped to reduce the non-exposed temperature of the GPCC. However, this rapid expansion caused the intumescent layer to be brittle and fail, so that, in this case, the hole formed and several layers of the expansion were detached from the GPCC (Figure 6). The intumescent structure for RHA-GPC 66.6:33.3 totally deteriorated, and the expansion collapsed, causing the temperature of the non-exposed part to rapidly increase until 568 °C at the 30th minute. Meanwhile, for both RHA-GPC 60:40 and RHA-GPC 70:30, even though the cracks caused the microstructure of the intumescent layer to collapse, no hole was formed on the burning point of the samples as the intumescent layer was still intact with the GPCC samples. 

The constant temperatures observed for all the RHA-GPC mix ratios except for RHA-GPC 66.6:33.3 were known as plateau temperatures. During these temperatures, the evaporation of water in the GPC occurred rapidly and was endothermic. A large amount of heat was absorbed due to the large latent heat of water evaporation present in the GPC structure, resulting in the more or less constant interface temperature of 90 °C. Unlike the RHA-GPC, where the plateau temperature occurred until the 60th minute of the testing, all the PKSA-GPC ratios only experienced a plateau temperature for a short period. The crack formation in the intumescent layer, as observed in Figure 6, allowed the heat and fire to penetrate through the GPC, resulting in a gradual increase in the non-exposed temperature of the PKSA-GPC after experiencing the plateau temperature. 

At the end of the fire test, the PKSA-GPC 70:30 demonstrated the lowest non-exposure temperature of 169 °C compared to the other PKSA-GPC mix ratios. Meanwhile, the RHA-GPC 70:30 exhibited the lowest non-exposure temperature of 93 °C compared to the others. These results showed that a high aluminosilicate content in the GPC contributed to better fire-retardant properties. Furthermore, based on the results obtained, it was shown that the RHA-GPC possessed a better thermal protective coating compared to the PKSA-GPC owing to the high molar ratio of Si/Al present in the RHA-GPC (Si/Al = 44.7 ± 5.36) compared to the PKSA-GPC (Si/Al = 30.37 ± 0.91), as shown in Figure 7. Moreover, a high Si/Al ratio resulted in a dense and highly homogenous microstructure, contributing to the high thermal conductivity of the GPC [49]. Finally, both the PKSA-GPC and RHA-GPC possessed better fire performance than the OPCC. These deductions were due to the presence of the stable and dense intumescent layer developed on the surface of the GPC mix ratios, which contributed to the better thermal stability of the GPC mix ratios compared to the OPCC.

#### 3.2.3. Thermal Expansion of the Geopolymer Concrete 

**Figure 10:**
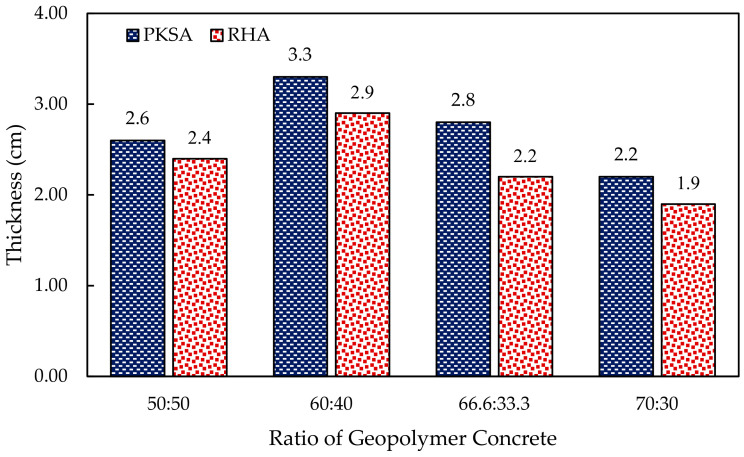
The expansion of the intumescent layer on the surface of the GPC.

Upon being subjected to high flame temperature, the GPC underwent an intumescent phenomenon where various components present in the GPC reacted and swelled when exposed to a high flame temperature. The formation of the intumescent layer on the concrete surface could provide protection to minimise the rise in temperature of the substance in the event of a fire. Figure 10 shows the expansion of the intumescent layer on the surface of the GPC with different mix ratios of PKSA-GPC and RHA-GPC.

Generally, Figure 10 shows that the expansion of the PKSA-GPC was higher than the RHA-GPC. Although the molar ratio of Si/Al in the RHA-GPC (Si/Al = 44.7 ± 5.36) was higher than the PKSA-GPC (Si/Al = 30.37 ± 0.91), the weak GPC matrix of RHA-GPC formed during the testing resulted in the collapse of the intumescent structure, as previously discussed in Section 3.2.1. Moreover, the collapse of the highly distributed pore network was likely to occur in the high molar ratio of Si/Al of the GPC [50]. Therefore, the PKSA-GPC formed a more compact and dense structure as compared to the RHA-GPC as it contained a lower Si/Al molar ratio than the RHA-GPC.

In addition, both PKSA-GPC and RHA-GPC, with a mix ratio of 60:40, revealed the highest expansion. In contrast, PKSA-GPC and RHA-GPC, with a mix ratio of 70:30, showed the lowest expansion. Based on the observations, the expansion did not show any distinct trend relating to the mix ratios of GPC with its expansion. Hence, there was no correlation between the thickness expansion with the increasing amount of ash in the GPC. 

The RHA-GPC 70:30 experienced the lowest expansion among the other mix ratios of PKSA-GPC and RHA-GPC, which might be due to the high amount of aluminosilicate present in the formulation. This was because an excessive amount of aluminosilicate could suppress the desirable expansion of the intumescent layer [51]. Conversely, the PKSA-GPC 60:40 demonstrated the best formulation with the highest thickness of the intumescent layer, which was 3.3 mm compared to the rest. This result indicated that this formulation had attained the optimal reaction in developing the densest layer when subjected to a high flame temperature. The optimum component compositions, such as the aluminosilicate source and the fine aggregates and their compatibility, could also significantly contribute to a high thermal expansion.

#### 3.2.4. Thermal Stability of the Geopolymer Concrete and OPCC

**Figure 11:**
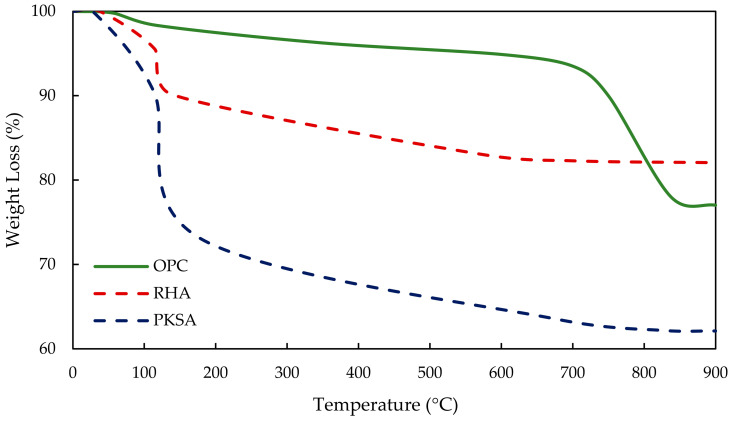
TGA thermogram of the OPCC and GPC.

The thermal stability of the GPC and OPCC was determined from the thermogravimetric analysis (TGA) conducted. The best formulations, PKSA-GPC 60:40, RHA-GPC 60:40 and OPCC, were further analysed using TGA, and the TGA thermogram is shown in Figure 11. This analysis measured the weight loss of the specimens while gradually being exposed to rising temperatures.

The TGA thermogram in Figure 11 shows a rapid decrease in weight loss for RHA-GPC 60:40 and PKSA-GPC 60:40 before the temperature of 200 °C. This phenomenon was due to the fast evaporation rate of water content in the GP matrix of PKSA-GPC 60:40 and RHA-GPC 60:40. This result also indicated that the intermolecular and crystalline water loss occurred in the GPC matrices [52]. Moreover, the dihydroxylation of hydroxyl in the Si-OH and Al-OH also largely contributed to moisture loss in the GPC matrices. Then, the RHA-GPC and PKSA-GPC experienced a minor weight loss after the rapid evaporation, suggesting that both mix ratios of GPC had achieved thermal stability.

On the other hand, the OPCC underwent a rapid weight loss after 700 °C, as observed in Figure 11. This weight loss indicated that OPCC experienced a massive loss of strength due to the loss of moisture content in its matrix from the dehydration of the hydration components, which was the calcium hydroxide present in the OPCC. Furthermore, the RHA-GPC and the PKSA-GPC experienced rapid evaporation before reaching the temperature of 200 °C. This phenomenon was because the heat travelled faster in the GPC compared to the OPCC. This phenomenon resulted in a low-temperature gradient in the geopolymer matrix [53]. Hence, the PKSA-GPC and the RHA-GPC achieved faster thermal stability compared to the OPCC. Next, it can be seen from Figure 11 that PKSA-GPC experienced the highest weight loss with a value of 37.88% compared to RHA-GPC (17.94%) and OPCC (23.99%). This result revealed that PKSA-GPC had the highest moisture content and volatile components compared to the RHA-GPC and OPCC.

### 3.3. GPC Microstructure Analysis

**Figure 12:**
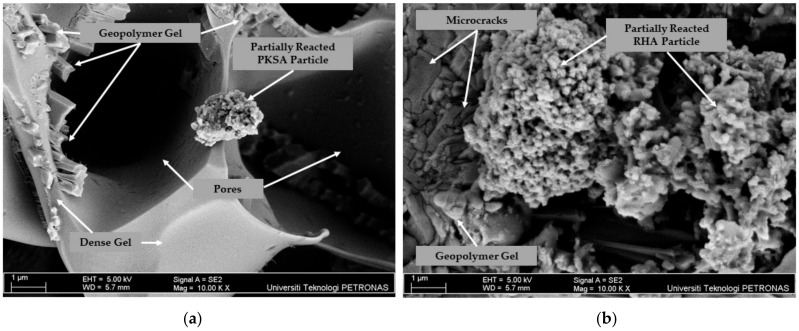
Surface SEM images of (**a**) PKSA-GPC 60:40 and (**b**) RHA-GPC 60:40.

**Figure 13:**
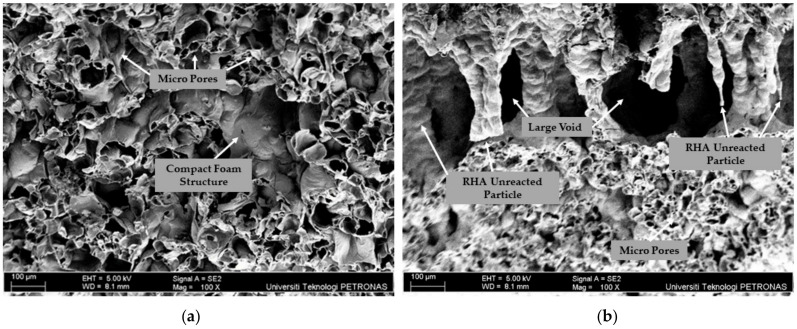
Cross-section SEM images of (**a**) PKSA-GPC 60:40 and (**b**) RHA-GPC 60:40.

The effectiveness of the GPC in providing fire protection is not solely determined by the thickness of the intumescent layer. The microstructure properties of the layer expansion also play a crucial role in reducing heat penetration. The microstructure properties of the best formulations PKSA-GPC 60:40 and RHA-GPC 60:40 were further analysed using SEM. Figure 12 and Figure 13 show the SEM images of the surface and a cross-sectional view of the intumescent layer for PKSA-GPC 60:40 and RHA-GPC 60:40, respectively.

Observation of the surface SEM image for PKSA-GPC 60:40 in Figure 12a displays the presence of a denser foam structure and a low amount of partially reacted PKSA particles. Not only that, most of the PKSA particles were activated and bonded with the aggregate, with an additional dense geopolymer gel observed. The PKSA particles, which have a larger specific surface area from particle size distribution analysis, formed an additional geopolymer gel. The dense geopolymer gel helped to fill the microcracks and created better cohesion between aggregates, leading to increased strength and creating a thermally stable intumescent layer. Moreover, in Figure 12b, it was apparent that a higher amount of RHA particles were not repolymerised or activated with the alkaline solution, and cracks were observed. The higher presence of partially reacted RHA particles led to an increase in extended cracks as the dense gel could not fill these voids and cracks effectively.

Upon observation, the cross-sectional SEM image for PKSA-GPC 60:40 in Figure 13 shows the presence of a compact foam structure, resulting in the formation of a stable layer. These highly dense and compact intumescent layers were created due to the low molar ratio of the Si/Al present in the PKSA-GPC 60:40, which contributed to a rigid three-dimensional polymer network [47]. Hence, the intumescent layers could develop effectively and be able to undergo proper crystallisation, creating a thermally stable intumescent layer. 

It can be clearly seen that the expansion of the intumescent layer in the cross-sectional SEM image of RHA-GPC 60:40 in Figure 13 exhibited a large empty void. These large voids can be attributed to the materials’ decomposition, which caused the layer to be brittle and crack on the surface of the RHA-GPC 60:40. The formations of these large empty voids were also due to the failure during the intumescent process caused by the high water content present in the RHA-GPC 60:40. As a rapid evaporation rate occurred, water molecules travelled from the exposed surface of the hot fire to the cooler inner part of the material, generating a large empty void in the middle of the intumescent layer. 

In conclusion, the PKSA-GPC 60:40 mixture demonstrated better fire performance compared to RHA-GPC 60:40 as the RHA-GPC underwent an incomplete intumescent and crystallisation process. The thermally stable compact intumescent layers of PKSA-GPC 60:40 effectively delayed the spread of fire for a longer duration compared to RHA-GPC 60:40. 

### 3.4. Drying Shrinkage of GPC

**Figure 14:**
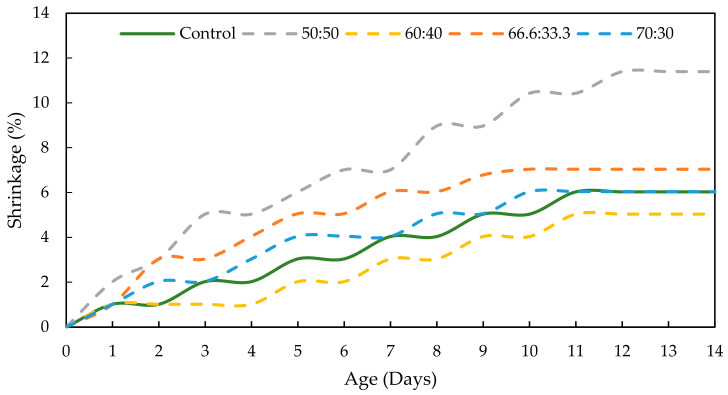
Effect of the ratios of GP to sand to the shrinkage of PKSA-GPC.

**Figure 15:**
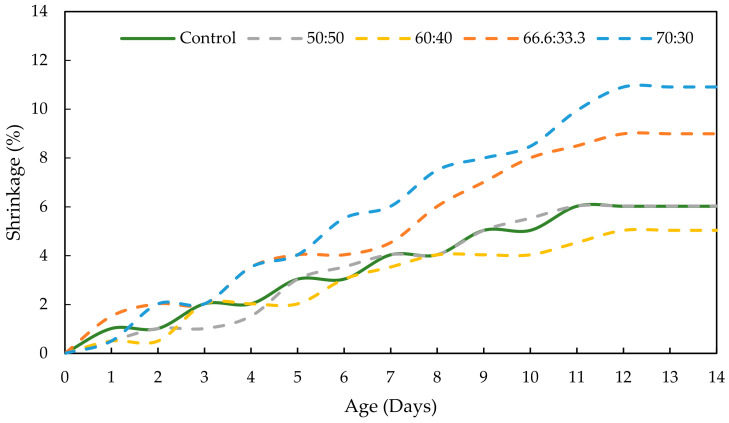
Effect of the ratios of GP to sand to the shrinkage of RHA-GPC.

Drying shrinkage was performed to determine the moisture loss in the GPC and OPCC structures. Figure 14 and Figure 15 present the shrinking of the OPCC, the PKSA-GPC and the RHA-GPC with different mix design ratios. The results presented a clear trend in which the shrinking of these concretes gradually increased with time due to the occurrence of water that escaped from the capillary pores of the OPCC and GPC to the unsaturated outside air. This then led to the volume contraction of the OPCC and GPC [15]. 

Based on Figure 14 and Figure 15, the RHA-GPC 60:40 and PKSA-GPC 60:40 exhibited better shrinking performance, where both underwent 5.040% shrinkage, than OPCC with a percentage of 8.996%. In contrast, PKSA-GPC 50:50 and RHA-GPC 70:30 showed the highest shrinkage among others, with percentages of 11.391% and 10.912%, respectively. These results revealed that the ratios of GP to sand did not influence the shrinkage of the GPC. Therefore, the increase in ash in the GPC did not affect the drying shrinkage of the GPC. These observations are consistent with the findings provided by Humad et al. (2019) [10]. In addition, the microcrack formation due to the contractions in the concrete’s volume could significantly contribute to the high shrinkage in the PKSA-GPC 50:50 and RHA-GPC 70:30 as the capillary water managed to escape from these microcracks. Hence, this event could explain the steep slope of the PKSA-GPC 50:50 and RHA-GPC 70:30, as observed in Figure 14 and Figure 15. On the other hand, the GPC with the mix ratio of 60:40 demonstrated the best shrinkage performance compared to the OPCC, later proving that the GPC with the mix ratios of 60 wt.% GP and 40 wt.% sand could be the optimum mix composition to minimise the drying shrinkage of the concrete. The determination of the drying shrinkage of concrete was crucial as it highly influenced the mechanical strength and durability of the concrete [54].

## 4. Conclusions

The PKSA-GPC was developed from the mixing of the PKSA into the GPC mixture with the addition of sand as its fine aggregate. This development underwent several preliminary tests to find the best mix designs. Four mix designs were selected based on their consistency, workable viscosity, as well as rheological and thixotropic behaviour. These mix designs were PKSA-GPC 70:30, PKSA-GPC 60:40, PKSA-GPC 50:50 and PKSA-GPC 50:25. Next, the performance of these mix designs was evaluated and compared with the RHA-GPC and OPCC.

- It was found that PKSA-GPC had better fire performance than the RHA-GPC and OPCC. The OPCC showed a rapid temperature increment when exposed to the high flame temperature, which increased from ambient temperature to 350 °C within 60 min of the flame exposure. In contrast, the highest temperature increment for the PKSA-GPC (PKSA-GPC 50:50) was 169 °C after being exposed to the high flame temperature for 60 min.
- Although the RHA-GPC possessed lower temperature increments when exposed to the high temperature from ambient temperature to 93 °C, the RHA-GPC formed holes due to the collapse of the intumescent layer, indicating that the intumescent layer was weaker than the PKSA-GPC. This was verified in the microstructural analysis, which showed that the PKSA-GPC has a dense and compact intumescent layer compared to the RHA-GPC.
- The molar ratio of the Si/Al played an important role in the formation of the intumescent layer as lower molar Si/Al contributed to a rigid three-dimensional polymer network, which caused a denser and more compact intumescent layer.
- Moreover, both the mix designs of RHA-GPC 60:40 and PKSA-GPC 60:40 showed better drying shrinkage performance with a drying shrinkage of 5.040% compared to the OPCC, which experienced 8.996% drying shrinkage.

In conclusion, PKSA-GPC can potentially be applied as a passive fire protection system owing to its ability to delay the spread of fire after ignition. It can also be an alternative to replace the conventional OPCC and RHA-GPC. Hence, the utilisation of the PKSA in GPC could help reduce the environmental problem that arises due to the improper disposal of the palm oil biomass.

## Figures and Tables

**Table 1:** Chemical composition (wt.%) of PKS and RHA.

Element	SiO₂	Al₂O₃	Fe₂O₃	CaO	K_2_O	TiO_2_	MgO	Others	LOI
PKSA	46.41	7.74	0.89	11.83	13.87	1.95	5.93	2.12	9.26
RHA	87.40	3.00	0.03	0.02	0.82	0.08	0.05	0.13	8.47

**Table 2:** Mix ratios for PKSA and RHA.

Ash	AA	NaOH	Na_2_SiO_3_
2	5	2	11

**Table 3:** Design of experiments for PKSA and RHA.

Mix	Ratio (wt. in Gram)					
	GP	Sand	PKSA/RHA	AA	NaOH	Na_2_SiO_3_
PKSA-GPC 40:60	80.00	120.00	22.86	57.14	8.78	48.36
RHA-GPC 40:60						
PKSA-GPC 50:50	100.00	100.00	28.57	71.43	10.99	60.44
RHA-GPC 50:50						
PKSA-GPC 60:40	120.00	80.00	34.29	85.71	13.19	72.52
RHA-GPC 60:40						
PKSA-GPC 66.6:33.3	133.33	66.67	38.09	95.24	14.65	80.59
RHA-GPC 66.6:33.3						
PKSA-GPC 70:30	140.00	60.00	40.00	100.00	15.38	84.62
RHA-GPC 70:30						
PKSA-GPC 80:20	160.00	40.00	45.71	114.29	17.58	96.71
RHA-GPC 80:20						
PKSA-GPC 90:10	180.00	20.00	51.43	128.57	19.78	108.79
RHA-GPC 90:10						

**Table 4:** Summary of the performance of the GPC mixture.

Ratio (wt.%)		Observation				
GP	Sand	Mixing of GP	Mixing of GP and Sand		During Specimen Casting	
		Thermodynamic	Thermodynamic	Consistency	Rheology	Thixotropy
90	10	Exothermic	Endothermic	High	✕	✕
80	20	Exothermic	Endothermic	High	✕	✕
70	30	Exothermic	Endothermic	High	✓	✓
66.6	33.3	Exothermic	Endothermic	High	✓	✓
60	40	Exothermic	Endothermic	High	✓	✓
50	50	Exothermic	Endothermic	High	✓	✓
40	60	Exothermic	Endothermic	Low	✕	✕

## Data Availability

All data are presented in the article.

## References

[1] Mehta P.K. (2004). High-performance, high-volume fly ash concrete for sustainable development. Proceedings of the International Workshop on Sustainable Development and Concrete Technology.

[2] Wałach D. (2020). Analysis of factors affecting the environmental impact of concrete structures. Sustainability.

[3] Kim T.H., Chae C.U. (2016). Environmental impact analysis of acidification and eutrophication due to emissions from the production of concrete. Sustainability.

[4] Teh S.H., Wiedmann T., Castel A., de Burgh J. (2017). Hybrid life cycle assessment of greenhouse gas emissions from cement, concrete and geopolymer concrete in Australia. J. Clean. Prod..

[5] Abdul Rashid M.K., Ramli Sulong N.H., Alengaram U.J. (2020). Fire resistance performance of composite coating with geopolymer-based bio-fillers for lightweight panel application. J. Appl. Polym. Sci..

[6] Suhendro B. (2014). Toward green concrete for better sustainable environment. Procedia Eng..

[7] Villaquirán-Caicedo M.A., de Gutiérrez R.M., Sulekar S., Davis C., Nino J.C. (2015). Thermal properties of novel binary geopolymers based on metakaolin and alternative silica sources. Appl. Clay Sci..

[8] So H.S. (2016). Spalling prevention of high performance concrete at high temperatures. High Performance Concrete Technology and Applications.

[9] Chen K., Wu D., Xia L., Cai Q., Zhang Z. (2021). Geopolymer concrete durability subjected to aggressive environments–A review of influence factors and comparison with ordinary Portland cement. Constr. Build. Mater..

[10] Humad A.M., Kothari A., Provis J.L., Cwirzen A. (2019). The effect of blast furnace slag/fly ash ratio on setting, strength, and shrinkage of alkali-activated pastes and concretes. Front. Mater. Sci..

[11] Ryu G.S., Lee Y.B., Koh K.T., Chung Y.S. (2013). The mechanical properties of fly ash-based geopolymer concrete with alkaline activators. Constr. Build. Mater..

[12] (2016). Concrete-Specification, Performance, Production and Conformity.

[13] Nikoloutsopoulos N., Sotiropoulou A., Kakali G., Tsivilis S. (2021). Physical and mechanical properties of fly ash based geopolymer concrete compared to conventional concrete. Buildings.

[14] Yang Y., Fang S., Feng W., Wan S., Li L., Tang Y. (2023). Flexural and compressive performance of BFRP-reinforced geopolymer sea-sand concrete beams and columns: Experimental and analytical investigation. Compos. Struct..

[15] Neupane K., Chalmers D., Kidd P. (2018). High-Strength Geopolymer Concrete- Properties, Advantages and Challenges. Adv. Mater..

[16] Fernández-Jiménez A., García-Lodeiro I., Palomo A. (2007). Durability of alkali-activated fly ash cementitious materials. J. Mater. Sci..

[17] Azarsa P., Gupta R. (2020). Durability and leach-ability evaluation of K-based geopolymer concrete in real environmental conditions. Case Stud. Constr..

[18] Chen Z., Yu J., Nong Y., Yang Y., Zhang H., Tang Y. (2023). Beyond time: Enhancing corrosion resistance of geopolymer concrete and BFRP bars in seawater. Compos. Struct..

[19] Lee W.H., Lin K.L., Chang T.H., Ding Y.C., Cheng T.W. (2020). Sustainable development and performance evaluation of marble-waste-based geopolymer concrete. Polymers.

[20] Onoja E., Attan N., Chandren S., Razak F.I.A., Keyon A.S.A., Mahat N.A., Wahab R.A. (2017). Insights into the physicochemical properties of the Malaysian oil palm leaves as an alternative source of industrial materials and bioenergy. Malays. J. Fund. Appl. Sci..

[21] Saba N., Jawaid M., Sultan M.T.H. (2017). Thermal properties of oil palm biomass based composites. Lignocellulosic Fibre and Biomass-Based Composite Materials: Processing, Properties and Applications.

[22] Adnan S.H., Azemi N.F.N.M., Osman M.H., Jeni M.L.A., Ern P.A.S., Yassin N.I.M., Akasyah W.M.N. (2019). Influence of Palm oil fuel ash (POFA) towards fire resistance performance of brick. J. Adv. Res. Fluid Mech. Therm. Sci..

[23] Hussin M.W., Abdullah K. (2009). Properties of palm oil fuel ash cement based aerated concrete panel subjected to different curing regimes. Malays. J. Civ. Eng..

[24] Jong L.Y., Teo D.C.L. (2014). Concrete Containing Palm Oil Fuel Ash (POFA) and Oil Palm Shell (OPS) Subjected to Elevated Temperatures. J. Civ. Eng. Sci. Technol..

[25] Sulaiman M.A., Muthusamy K., Aris S.M., Rasid M.M., Paramasivam R., Othman R. (2018). Effect of unground oil palm ash as mixing ingredient towards properties of concrete. IOP Conference Series: Earth and Environmental Science.

[26] Tam V.W., Le K.N., Evangelista A.C.J., Butera A., Tran C.N., Teara A. (2019). Effect of fly ash and slag on concrete: Properties and emission analyses. Front. Eng. Manag..

[27] He P., Wang M., Fu S., Jia D., Yan S., Yuan J., Zhou Y. (2016). Effects of Si/Al ratio on the structure and properties of metakaolin based geopolymer. Ceram. Int..

[28] (2020). Standard Specification for Woven Wire Test Sieve Cloth and Test Sieves.

[29] Verma M., Dev N. (2022). Effect of liquid to binder ratio and curing temperature on the engineering properties of the geopolymer concrete. Silicon.

[30] Abdullah M.N., Mustapha M., Sallih N., Ahmad A., Mustapha F., Dahliyanti A. (2021). Study and use of rice husk ash as a source of aluminosilicate in refractory coating. Materials.

[31] Amran M., Debbarma S., Ozbakkaloglu T. (2021). Fly ash-based eco-friendly geopolymer concrete: A critical review of the long-term durability properties. Constr. Build. Mater..

[32] Gupta N., Gupta A., Saxena K.K., Shukla A., Goyal S.K. (2021). Mechanical and durability properties of geopolymer concrete composite at varying superplasticizer dosage. Mater. Today Proc..

[33] (2019). Standard Test Methods for Fire Tests of Building Construction and Materials.

[34] (2016). Standard Test Method for Linear Drying Shrinkage of Concrete Masonry Units.

[35] Xu H., Van Deventer J.S.J. (2000). The geopolymerisation of alumino-silicate minerals. Int. J. Miner. Process..

[36] Provis J.L. (2006). Modelling the Formation of Geopolymers Modelling the Formation of Geopolymers. Ph.D. Dissertation.

[37] Bosenick A., Dove M.T., Myers E.R., Palin E.J., Sainz-Diaz C.I., Guiton B.S., Redfern S.A.T. (2001). Computational methods for the study of energies of cation distributions: Applications to cation-ordering phase transitions and solid solutions. Miner. Mag..

[38] Ling Y., Wang K., Wang X., Hua S. (2019). Effects of mix design parameters on heat of geopolymerization, set time, and compressive strength of high calcium fly ash geopolymer. Constr. Build. Mater..

[39] Mohamed R., Abd Razak R., Abdullah M.M.A.B., Shuib R.K., Mortar N.A.M., Zailani W.W.A. (2019). . Investigation of heat released during geopolymerization with fly ash based geopolymer. IOP Conference Series: Materials Science and Engineering.

[40] Nodehi M., Taghvaee V.M. (2022). Alkali-activated materials and geopolymer: A review of common precursors and activators addressing circular economy. Circ. Econ. Sustain..

[41] Autef A., Joussein E., Gasgnier G., Rossignol S. (2012). Role of the silica source on the geopolymerization rate. J. Non-Cryst..

[42] Bian H., Hannawi K., Takarli M., Molez L., Prince W. (2016). Effects of thermal damage on physical properties and cracking behavior of ultrahigh-performance fiber-reinforced concrete. J. Mater. Sci..

[43] Hager I. (2013). Behaviour of cement concrete at high temperature. Bull. Pol. Acad. Sci. Tech..

[44] Fletcher I.A., Welch S., Torero J.L., Carvel R.O., Usmani A. (2017). Behaviour of concrete structures in fire. Therm. Sci..

[45] Sakkas K., Panias D., Nomikos P., Sofianos A. (2017). Comparison of fire resistant geopolymers for passive fire protection of concrete tunnel linings. Open Access Libr. J..

[46] Abdullah M.N., Mustapha F., Ahmad K.A., Mustapha M., Khan T., Singh B., Sebaey T.A. (2022). Effect of different pre-treatment on the microstructure and intumescent properties of rice husk ash-based geopolymer hybrid coating. Polymers.

[47] Abdullah M.M.A., Hussin K., Bnhussain M., Ismail K.N., Ibrahim W.M.W. (2011). Mechanism and chemical reaction of fly ash geopolymer cement—A review. Int. J. Pure Appl. Sci. Technol..

[48] Nijland T.G., Larbi J.A. (2010). Microscopic examination of deteriorated concrete. Non-Destructive Evaluation of Reinforced Concrete Structures.

[49] He R., Dai N., Wang Z. (2020). Thermal and mechanical properties of geopolymers exposed to high temperature: A literature review. Adv. Civ. Eng..

[50] Lahoti M., Wong K.K., Yang E.H., Tan K.H. (2018). Effects of Si/Al molar ratio on strength endurance and volume stability of metakaolin geopolymers subject to elevated temperature. Ceram. Int..

[51] Beh J.H., Yew M.C., Saw L.H., Yew M.K. (2020). Fire resistance and mechanical properties of intumescent coating using novel bioash for steel. Coatings.

[52] Baykara H., Cornejo M.H., Espinoza A., García E., Ulloa N. (2020). Preparation, characterization, and evaluation of compressive strength of polypropylene fiber reinforced geopolymer mortars. Heliyon.

[53] Sarker P.K., Kelly S., Yao Z. (2014). Effect of fire exposure on cracking, spalling and residual strength of fly ash geopolymer concrete. Mater. Des..

[54] Liew K.M., Sojobi A.O., Zhang L.W. (2017). Green concrete: Prospects and challenges. Constr. Build. Mater..

